# Efficacy of Rituximab Versus Cyclophosphamide and Mycophenolate for the Treatment of Interstitial Lung Disease in Systemic Sclerosis: A Systematic Review

**DOI:** 10.7759/cureus.68279

**Published:** 2024-08-31

**Authors:** Aneri Parikh, Aida J Francis, Chithra Sreenivasan, Manorama Pandey, Osamah AlQassab, Tatchaya Kanthajan, Esraa M AlEdani

**Affiliations:** 1 Internal Medicine, California Institute of Behavioral Neurosciences & Psychology, Fairfield, USA; 2 Dermatology, California Institute of Behavioral Neurosciences & Psychology, Fairfield, USA

**Keywords:** systemic sclerosis, administration and dosage, toxicity, adverse effects, interstitial lung disease, mycophenolate, rituximab, cyclophosphamide

## Abstract

Interstitial lung disease (ILD) is a common complication of systemic sclerosis (SSc), contributing to significant morbidity and mortality in affected individuals. The optimal treatment approach for SSc-associated ILD remains uncertain, with rituximab, cyclophosphamide, and mycophenolate among potential therapeutic options. This systematic review aims to evaluate and synthesize the existing evidence on the efficacy of rituximab compared to cyclophosphamide and mycophenolate for the treatment of ILD in patients with systemic sclerosis. A comprehensive search of the following electronic databases, PubMed, Science Direct, Google Scholar, and Cochrane Library, has been conducted to identify relevant studies, including randomized controlled trials, systematic review and meta-analysis, prospective cohort studies, and retrospective cohort studies. Data on study characteristics, participant demographics, interventions, outcomes, and key findings have been extracted and synthesized. The risk of bias in the included studies has been assessed using appropriate tools such as the Cochrane Bias assessment tool for randomized controlled trials, the New Castle Ottawa tool for cohort studies, and the AMSTAR checklist for systematic reviews and meta-analysis. The research team ultimately selected 15 high-quality studies for review. Rituximab demonstrated similar efficacy to cyclophosphamide and mycophenolate in improving lung function (forced vital capacity (FVC) and diffusing capacity of the lung for carbon monoxide (DLCO)), with fewer severe adverse events. Cyclophosphamide, while effective, had higher toxicity, leading to more frequent adverse events such as leukopenia and infections. Mycophenolate showed comparable efficacy to cyclophosphamide but with fewer side effects, making it a well-tolerated alternative. The findings of this systematic review will provide valuable insights into the comparative efficacy of rituximab, cyclophosphamide, and mycophenolate in the management of ILD in systemic sclerosis, informing clinical decision-making and guiding future research in this area.

## Introduction and background

Systemic sclerosis (SSc), often referred to as scleroderma, is a persistent autoimmune condition marked by the thickening and scarring of the skin and internal organs, notably the lungs [1,2]. Interstitial lung disease (ILD) is a prevalent lung complication in SSc, affecting up to 70% of individuals and considerably influencing their quality of life and prognosis. Managing SSc-related ILD is clinically challenging due to its varied presentation and inconsistent treatment responses [3-5].

Rituximab, a monoclonal antibody targeting CD20-positive B cells, has emerged as a potential therapeutic agent for SSc-associated ILD, with some studies suggesting beneficial effects on lung function and disease progression. However, the comparative efficacy of rituximab versus conventional immunosuppressive agents such as cyclophosphamide and mycophenolate remains unclear [6,7].

Cyclophosphamide, a cytotoxic agent, has been used for decades in the treatment of autoimmune diseases, including SSc-associated ILD. Mycophenolate, a selective immunosuppressant, has also shown promise in the management of ILD in SSc patients. Despite their widespread use, limited evidence exists regarding their comparative efficacy and safety profile compared to rituximab [8-10].

Given the lack of consensus regarding the optimal treatment approach for SSc-associated ILD, there is a critical need to systematically evaluate and synthesize the existing evidence on the efficacy of rituximab, cyclophosphamide, and mycophenolate. This systematic review aims to address this gap in knowledge, providing clinicians and researchers with valuable insights into the comparative effectiveness of these therapeutic agents for the treatment of ILD in SSc.

## Review

Methods

Search Strategy

A PICO (Population, Intervention, Comparison, and Outcome) was formulated in this systematic review to provide a structured framework for guiding the methodology section. The primary focus of the investigation (I) is treatment with rituximab. This will be compared (C) to the treatment with cyclophosphamide and/or mycophenolate. The primary outcome (O) includes improvement or stabilization in lung function, specifically changes in forced vital capacity (FVC) and diffusing capacity of the lungs for carbon monoxide (DLCO). Secondary outcomes include survival rates, quality of life measures, adverse events and safety profiles, modified Rodnan skin score (mRSS) comparison, and markers of disease progression (e.g., high-resolution CT findings). The present systematic review adheres to the PRISMA (Preferred Reporting Items for Systematic Reviews and Meta-Analyses) guidelines.

A comprehensive search was conducted in PubMed, Science Direct, Google Scholar, and Cochrane Library databases using keywords and MeSH terms related to "rituximab," "cyclophosphamide," "mycophenolate," "interstitial lung disease," "adverse effects," "toxicity," "administration and dosage," and "systemic sclerosis." The search was limited to studies published in English, as shown in Table 1 below.

**Table 1:** Search strategy

Search Engine	Search Strategy	Papers Identified	Picking Relevant Titles	Post-scanning Relevant Abstract	Post-quality Check
PubMed	General Field Search + MeSH and advanced search combined with filters)	37	26	20	7
Google Scholar	General Field Search with Boolean term “AND”	33	17	13	8
Science Direct	General Field Search with Boolean term “AND” combined with database filters	24	13	4	0
Cochrane Library	Regular Search	9	5	2	0
Total		103	61	39	15

Inclusion Criteria

The inclusion criteria for this review consisted of studies that compared the efficacy of rituximab versus cyclophosphamide and/or mycophenolate in treating ILD in patients with SSc. Eligible studies included randomized controlled trials (RCTs), prospective cohort studies, and retrospective cohort studies. The studies had to include adult patients diagnosed with SSc and ILD and report relevant outcomes such as changes in lung function parameters (e.g., FVC, DLCO), radiological findings, clinical outcomes (e.g., mortality, exacerbation rate), and adverse events. Only studies published in English and subjected to peer review were included.

Exclusion Criteria

The exclusion criteria for this review included case reports, case series, reviews, and conference abstracts. Studies involving pediatric populations or patients with connective tissue diseases other than SSc were also excluded. Additionally, studies with inadequate or incomplete data, as well as those published in languages other than English, were not considered for inclusion.

Quality Assessment

The search results were transferred into EndNote (Clarivate, Philadelphia, Pennsylvania) and transformed into a Microsoft Excel file (Microsoft Corporation, Redmond, Washington) as a second step. The duplicate papers were then removed using the duplicate filter. After analyzing the article titles, relevant titles were retained on a separate sheet.

**Figure 1:**
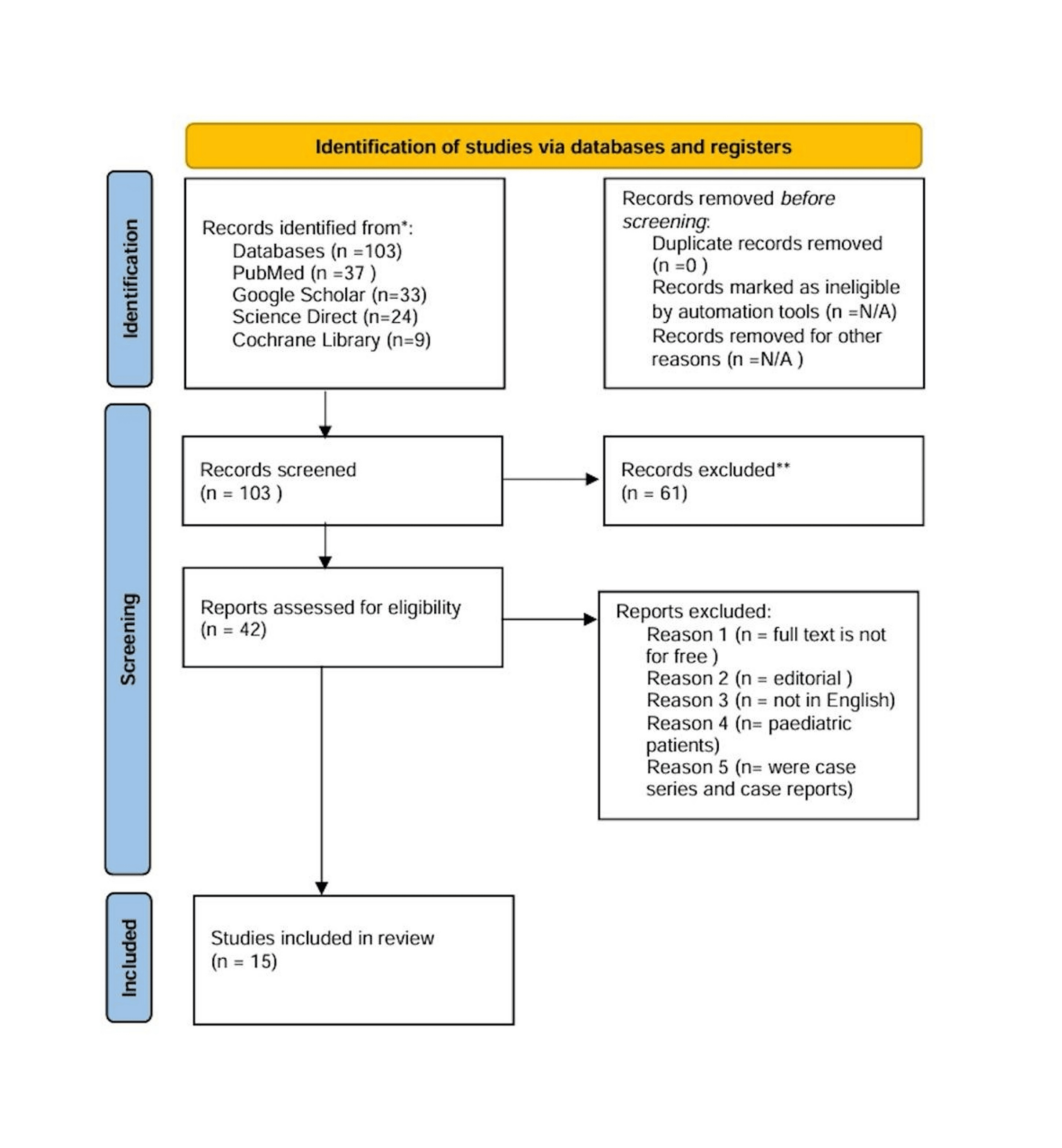
PRISMA flowchart

Each article was analyzed individually for quality appraisal and the potential risk of bias. Three assessment tools were used to check the quality of the included papers. Systematic reviews were evaluated using the Assessment of Multiple Systematic Reviews (AMSTAR 2), cohort studies were assessed using the Newcastle-Ottawa Scale, and the RCTs were subjected to the Cochrane risk-of-bias assessment tool scale for RCTs (Tables 2, 3, 4). The articles were scored as either high quality, low quality, or unclear, and only 70% of articles achieving the quality assessment tool were included, as shown in the PRISMA chart below (Figure 1).

**Table 2:** Newcastle-Ottawa Scale for observational studies (+) indicates a low-quality score in the respective category; (++) indicates a moderate-quality score; (+++) indicates a high-quality score; (++++) indicates a very high-quality score

Study	Selection	Comparability	Outcome
Lepri et al. (2016) [11]	+++	++	+++
Fitzgerald et al. (2015) [12]	+++	++	++
Moreno-Torres et al. (2023) [13]	++++	++	+++
Narvaez et al. (2020) [14]	++++	++	+++
Yilmaz et al. (2021) [9]	+++	+	+++
Elhai et al. (2018) [15]	++++	++	+++
Zhu et al. (2020) [16]	+++	++	+++

**Table 3:** AMSTAR 2 checklist for systematic review and meta-analysis [19] RoB: risk-of-bias tool for randomized trials; PICO: patient/population, intervention, comparison, and outcomes; RCT: randomized controlled trial; NRSI: non-randomized studies of interventions; AMSTAR: Assessment of Multiple Systematic Reviews

Questions	Barnes et al. (2024) [8]	Erre et al. (2020) [1]	Macrea et al. (2024) [7]	Herman et al. (2024) [17]	Xinyu Ma et al. (2021) [18]
Did the research questions and inclusion criteria for the review include the components of PICO?	Yes	Yes	Yes	Yes	Yes
Did the report of the review contain an explicit statement that the review methods were established prior to the conduct of the review and did the report justify any significant deviations from the protocol?	Yes	Yes	Yes	Yes	Yes
Did the review authors explain their selection of the study designs for inclusion in the review?	Yes	Yes	Yes	Yes	Yes
Did the review authors use a comprehensive literature search strategy?	Yes	Yes	Yes	Yes	Yes
Did the review authors perform study selection in duplicate?	Yes	Yes	Yes	Yes	Yes
Did the review authors perform data extraction in duplicate?	Yes	Yes	Yes	Yes	Yes
Did the review authors provide a list of excluded studies and justify the exclusions?	Yes	Yes	Yes	Yes	Yes
Did the review authors describe the included studies in adequate detail?	Yes	Yes	Yes	Yes	Yes
Did the review authors use a satisfactory technique for assessing the risk of bias (RoB) in individual studies that were included in the review?	RCT - Yes NRSI - Yes	RCT - Yes NRSI - Yes	RCT - Yes NRSI - Yes	RCT - Yes NRSI - Yes	RCT - Yes NRSI - Yes
Did the review authors report on the sources of funding for the studies included in the review?	Yes	Yes	Yes	Yes	Yes
If meta-analysis was performed did the review authors use appropriate methods for statistical combination of results?	RCT - Yes NRSI - Yes	RCT - Yes NRSI - Yes	RCT - Yes NRSI - Yes	RCT - Yes NRSI - Yes	RCT - Yes NRSI - Yes
If meta-analysis was performed, did the review authors assess the potential impact of RoB in individual studies on the results of the meta-analysis or other evidence synthesis?	Yes	Yes	Yes	Yes	Yes
Did the review authors account for RoB in individual studies when interpreting/discussing the results of the review?	Yes	Yes	Yes	Yes	Yes
Did the review authors provide a satisfactory explanation for, and discussion of, any heterogeneity observed in the results of the review?	Yes	Yes	Yes	Yes	Yes
If they performed quantitative synthesis did the review authors carry out an adequate investigation of publication bias (small study bias) and discuss its likely impact on the results of the review?	Yes	Yes	Yes	Yes	Yes
Did the review authors report any potential sources of conflict of interest, including any funding they received for conducting the review?	Yes	Yes	Yes	Yes	Yes

**Table 4:** Revised Cochrane risk-of-bias tool for randomized controlled trials (RoB2). Each RCT was evaluated for bias, receiving a rating of "Low," "High," or "Some concerns" [21]

Selected Study	Bias Arising From the Randomization Process	Bias Due to Deviations From the Intended Intervention	Bias Due to Missing Outcome Data	Bias in the Measurement of the Outcome	Bias in the Selection of the Report
Maher et al. (2023) [6]	Low	Low	Low	Low	Low
Sircar et al. (2018) [10]	Low	Low	Low	Low	Low
Mankikian et al. (2022) [20]	Low	Some concern	Some concern	Low	Low

Results

Our systemic research yielded a total of 103 papers, and after detailed article screening, 15 articles were included in our study. Out of these, three RCTs, five systematic reviews and meta-analysis, six retrospective observational comparative cohort studies, and one prospective cohort study were included in this study. Across all included studies, 1728 patients were identified and included. Participants were adults with confirmed SSc-ILD, and interventions included rituximab, cyclophosphamide, and mycophenolate. Baseline characteristics such as age, gender, disease duration, and baseline lung function were comparable across the studies (Table 5).

**Table 5:** Summary of findings ILD: interstitial lung disease; SYN: synovitis; MCTD: connective tissue disorder; SSc: systemic sclerosis; DLCO: diffusing capacity of carbon monoxide; FVC: functional vital capacity; MMF: mycophenolate mofeti; PFS: progression-free survival; CI: confidence interval; RTX: rituximab; CYC: cyclophosphamide

Sr. No.	Author	Publication Year	Type of Study	Patient Population	Outcome/Result
1.	Lepri et al. [11]	2016	Retrospective cohort study	A retrospective analysis involving multiple centers examined patients with ILD secondary to SYN (n=15), MCTD (n=6), and SSc (n=23).	A retrospective cohort study was conducted across multiple centers, analyzing patients with interstitial lung disease (ILD) secondary to synovitis (SYN) (n=15), mixed connective tissue disease (MCTD) (n=6), and systemic sclerosis (SSc) (n=23). The median forced vital capacity (FVC) for the entire cohort changed from 53.0% (42.0-90.0) at baseline to 51.4% (45.6-85.0) after 1 year and 63.0% (50-88) after 2 years (p=0.14). Specifically, in SSc patients, FVC shifted from 81.0% (66.0-104.0) at baseline to 89.0% (65.0-113.0) at 1 year (p=0.1) and 74.5% (50-91) after 2 years (p=0.07). For the MCTD group, FVC altered from 64.5% (63.0-68.0) at baseline to 63.0% (59.0-71.0) at 1 year (p=0.6) and 61% (59-71) after 2 years (p=0.8). The diffusing capacity for carbon monoxide (DLCO) showed a trend towards improvement in the SYN population (p=0.06 at 1 year and 0.2 at 2 years), while changes remained non-significant in the SSc and MCTD groups. Among SYN patients, 33.3% responded to treatment after 1 year for FVC, compared to 9.5% in SSc (p=0.07) and 17% in MCTD (p=0.45).
2.	Fitzgerald et al. [12]	2015	Retrospective Cohort study	Ten patients who underwent treatment with RTX for pulmonary complications of CTD were identified. Baseline demographics, pre- and post-treatment assessments, and adverse events were documented over an average follow-up period of 12.3 months (range: 3 to 27 months).	There was an average increase of 19% in DLCO (median DLCO in ml/min/mmHg before and after treatment: 13.94 vs. 19.34, p=0.028) and a mean rise of 13% in FVC (median FVC in liters before and after treatment: 3.47 vs. 3.6, p=0.28). Among patients with pulmonary fibrosis (n=7), the CT scans showed an improvement in severity post-treatment, although this improvement was not statistically significant. Additionally, follow-up scans for two patients without fibrosis showed a reduction in the number of nodules. Importantly, no patient experienced a severe adverse reaction to RTX.
3.	Barnes et al. [8]	2024	Systematic review and Meta-Analysis	Five studies were incorporated into the review: two randomized controlled trials comparing cyclophosphamide against placebo, and one randomized controlled trial alongside two retrospective case-control studies comparing cyclophosphamide with mycophenolate.	Compared to the placebo, cyclophosphamide resulted in a 2.83% reduction in the decline of predicted forced vital capacity (FVC) over 12 months (95% confidence interval (CI), 0.80-4.87; low evidence). There were notable improvements in breathlessness (Transition Dyspnea Index mean difference (MD), 2.90; 95% CI, 1.94-3.86; minimum clinically important difference, 1; moderate evidence) and in disability (Health Assessment Questionnaire-Disability Index MD, -0.16; 95% CI, -0.28 to -0.04; -0.14; moderate evidence). Cyclophosphamide use was associated with higher risks of leukopenia and general symptoms, but no difference in mortality was observed. When comparing cyclophosphamide to mycophenolate, the latter showed better results in the diffusing capacity of the lung for carbon monoxide % predicted at 6 months (MD, -3.67%; 95% CI, -6.3% to -1.1% unadjusted; MD, -4.88%; 95% CI, -7.3% to -2.5% adjusted for alveolar volume; moderate evidence), 12 months (MD, -5.90%; 95% CI, -8.4% to -3.4% adjusted for alveolar volume; moderate evidence), and 18 months (MD, -3.26%; 95% CI, -6.1% to -0.4%; moderate evidence), but not at 24 months. There were no significant differences in FVC % predicted, mortality, or quality-of-life outcomes between the two drugs. However, participants were more likely to discontinue cyclophosphamide prematurely compared to mycophenolate (relative risk, 1.70; 95% CI, 1.10-2.63; high-certainty evidence).
4.	M Maher et al. [6]	2023	Randomized Control trial	Patients aged 18-80 years with severe or progressive ILD associated with scleroderma, idiopathic inflammatory myositis, or mixed connective tissue disease were recruited from 11 specialized ILD or rheumatology centers in the UK. They were randomly assigned in a 1:1 ratio to receive either rituximab (1000 mg at weeks 0 and 2 intravenously) or cyclophosphamide (600 mg/m2 body surface area every 4 weeks intravenously for six doses). A total of 145 participants were screened, and 101 were randomly allocated: 50 (50%) to the cyclophosphamide group and 51 (50%) to the rituximab group. In the analysis, 48 (96%) participants in the cyclophosphamide group and 49 (96%) in the rituximab group received at least one dose of treatment. Of these, 43 (86%) participants in the cyclophosphamide group and 42 (82%) participants in the rituximab group completed 24 weeks of treatment and follow-up.	At 24 weeks, both the cyclophosphamide group and the rituximab group showed improvements in FVC from baseline, with an unadjusted mean increase of 99 mL (SD 329) in the cyclophosphamide group and 97 mL (234) in the rituximab group. In the adjusted mixed-effects model, the difference in the primary endpoint at 24 weeks was -40 mL (95% CI -153 to 74; p=0.49) between the rituximab and cyclophosphamide groups. KBILD quality-of-life scores increased by a mean of 9.4 points (SD 20.8) in the cyclophosphamide group and 8.8 points (17.0) in the rituximab group at 24 weeks. All participants experienced at least one adverse event during the study, with numerically fewer adverse events reported in the rituximab group (445 events) compared to the cyclophosphamide group (646 events).
5.	Erre et al. [1]	2020	Systematic review and network Meta-Analysis	Nine randomized clinical trials involving 926 participants comparing eight different interventions and placebo, with an average follow-up of one year, were included in the analysis.	Rituximab, unlike the placebo, significantly reduced the decline in FVC (SMD (95% CI) = 1.00 (0.39 to 1.61)). There were no significant differences in safety and mortality across the treatments and placebo, although the number of reported events was low. Cyclophosphamide and pomalidomide were less well-tolerated compared to the placebo, mycophenolate, and nintedanib.
6.	Moreno-Torres et al. [13]	2023	Retrospective Observational Comparative Study	In total, 47 patients were enrolled: 22 (47%) in the CYC group and 25 (53%) in the non-CYC group, comprising 32% on azathioprine, 28% on glucocorticoids alone, 20% on mycophenolate, 16% on calcineurin inhibitors and methotrexate, and 4% on rituximab. The majority (81%) of patients were female, with a mean age of 50.4 years.	FVC improvement was seen in 64% of patients in the CYC group compared to 32% in the non-CYC group (p = 0.03). In the logistic regression model, CYC emerged as the sole independent predictor of FVC improvement (OR=3.97, 95% CI 1.07-14.75). Patients in the CYC group gained more methyl-prednisolone pulses (59% vs. 28% in the non-CYC group, p = 0.03), fewer initial doses of GCs over 30 mg/day (19% vs. 77%, p = 0.001), and had lower average doses of prednisone over six months (11 mg/day vs. 31.1 mg/day, p = 0.001).
7.	Narváez et al. [14]	2020	Retrospective Observational Study	Twenty-four patients were included.	Prior to starting RTX, the mean decline in %pFVC and %pDLCO over the previous two years was -12.9% and -12.5%, respectively. After one year of RTX treatment, there was a significant improvement in %pFVC (∆+8.8% compared to baseline, 95% CI: -13.7 to -3.9; p = 0.001) and %pDLCO (∆+4.6%, 95% CI: -8.2 to -0.8; p = 0.018). Additionally, there was a notable reduction in the median prednisone dose, with 25% of patients able to discontinue it. After two years of treatment, RTX had been discontinued in nine patients (three due to adverse events and six due to inefficacy). In the 15 patients (62.5%) who completed 24 months of therapy, the significant improvements in pulmonary function test parameters were maintained: ∆%pFVC: +11.1% (95% CI: -17.6 to -4.5; p = 0.003) and ∆%pDLCO: +8.7% (95% CI: -13.9 to -8.3; p = 0.003).
8.	Macrea et al. [7]	2024	Systematic review and Meta-Analysis	A systematic review was undertaken across the MEDLINE, EMBASE, and Cochrane Central Register of Controlled Trials (CENTRAL) databases up to June 2022 to identify studies investigating the use of rituximab in the treatment of systemic sclerosis-associated interstitial lung disease (SSc-ILD). Three pertinent studies meeting the criteria were included in the review.	Rituximab notably enhanced the forced vital capacity % predicted (mean difference of 3.13; 95% confidence interval (CI), 0.37 to 5.90) and reduced the modified Rodnan Skin Score (mean difference of -7.01; 95% CI, -11.46 to -2.56) over 24–48 weeks.
9.	Herman et al. [17]	2024	Systematic review and Meta-Analysis	A search of the MEDLINE, EMBASE, and CENTRAL databases was conducted up to June 2022 to identify studies utilizing mycophenolate for the treatment of patients with systemic sclerosis-associated interstitial lung disease (SSc-ILD). Seven studies meeting the inclusion criteria were identified from the literature review.	The systematic review and meta-analyses indicated favorable changes in forced vital capacity % predicted (mean difference (MD), 5.4%; 95% confidence interval (95% CI): 3.3%, 7.5%), diffusing capacity of the lung for carbon monoxide % predicted (MD, 4.64%; 95% CI: 0.54%, 8.74%), and breathlessness score (MD, 1.99; 95% CI: 0.36, 3.62) with mycophenolate compared to placebo. Mycophenolate showed a higher risk of anemia (relative risk (RR), 2.3; 95% CI: 1.2, 71.4). There were no significant differences between mycophenolate and cyclophosphamide, except for a lower risk of premature discontinuation (RR, 0.6; 95% CI: 0.4, 0.9) and leukopenia (RR, 0.1; 95% CI: 0.05, 0.4) favoring mycophenolate.
10.	Yılmaz et al. [9]	2021	Observational Cohort Study	Symptoms and respiratory function parameters were compared between 34 patients treated with cyclophosphamide and 27 patients treated with rituximab for a minimum of 24 months.	Symptoms such as cough, Raynaud’s phenomenon, digital ulceration, diarrhea, and dysphagia showed statistically significant improvement in the rituximab group (p = 0.004, p = 0.001, p = 0.006, p = 0.005).
11.	Xinyu Ma et al. [18]	2021	Systematic Review and Meta-Analysis	Six studies that fulfilled the inclusion criteria (comprising one randomized controlled trial, three prospective observational studies, and two retrospective observational studies) were selected.	The weighted mean difference (WMD) of FVC change between the MMF and CYC groups was -1.17 (95% CI: -2.713 to 0.373; P = 0.137), indicating a non-significant difference favoring MMF. For DLco change, the summary WMD in the MMF group compared to the CYC group was 2.245 (95% CI: 0.258 to 4.232; P = 0.027), showing a statistically significant improvement favoring MMF. Studies also reported fewer adverse events in the MMF group compared to CYC.
12.	Sircar et al. [10]	2018	Randomized Control trial	Sixty systemic sclerosis (SSc) patients aged between 18 and 60 years, presenting with both skin and lung involvement, were randomly assigned to receive either monthly pulses of cyclophosphamide (CYC) at 500 mg/m2 or two doses of rituximab (RTX) at 1000 mg each, administered at 0 and 15 days.	The mean FVC (% (standard deviation)) in the RTX group improved from 61.30 (11.28) to 67.52 (13.59), whereas in the CYC group, it declined from 59.25 (12.96) to 58.06 (11.23) at 6 months (P = 0.003). The absolute change in FVC was 1.51 (0.45) L to 1.65 (0.47) L in the RTX group, compared to 1.42 (0.49) L to 1.42 (0.46) L in the CYC group. The modified Rodnan Skin Score (mRSS) decreased from 21.77 (9.86) to 12.10 (10.14) in the RTX group and from 23.83 (9.28) to 18.33 (7.69) in the CYC group after 6 months. Serious adverse events were more frequently reported in the CYC group.
13.	Mankikian et al. [20]	2022	A double-blind, randomized, placebo controlled trial	122 randomized patients received at least one dose of rituximab (n=63) or placebo (n=59).	The least-squares mean change in FVC (% predicted) from baseline to 6 months was +1.60 (standard error (SE) 1.13) in the rituximab plus MMF group and -2.01 (SE 1.17) in the placebo plus MMF group (difference between groups 3.60, 95% CI 0.41–6.80; p=0.0273). Progression-free survival (PFS) was superior in the rituximab plus MMF group (crude hazard ratio 0.47, 95% CI 0.23–0.96; p=0.03). Serious adverse events occurred in 26 (41%) patients in the rituximab plus MMF group and in 23 (39%) in the placebo plus MMF group. Nine infections were reported in the rituximab plus MMF group (five bacterial infections, three viral infections, one other) compared to four bacterial infections in the placebo plus MMF group.
14.	Elhai et al. [15]	2018	Prospective Cohort study	254 patients were included.	Comparison of treated patients with 9575 propensity-score matched patients indicated that individuals treated with rituximab were more likely to experience improvement in skin fibrosis (22.7 vs 14.03 events per 100 person-years; odds ratio (OR): 2.79 (1.47–5.32); p=0.002). Treated patients did not show significantly different rates of decline in forced vital capacity (FVC) >10% (OR: 1.03 (0.55–1.94); p=0.93) or carbon monoxide diffusing capacity (DLCO) decrease. Patients who received rituximab were more likely to discontinue or reduce steroid use (OR: 2.34 (1.56–3.53), p<0.0001). Patients treated concurrently with mycophenolate mofetil showed a trend towards better outcomes compared to those receiving rituximab alone (delta FVC: 5.22 (0.83–9.62); p=0.019 compared to controls vs 3 (0.66–5.35); p=0.012).
15.	Zhu et al. [16]	2020	Retrospective Cohort study	This retrospective study included 83 patients.	The median duration of interstitial lung disease (ILD) at the initiation of treatment was longer in the rituximab group at 47 months (range: 4-170) compared to 6.5 months (range: 0-164) in the control group. After treatment, forced vital capacity (FVC) decreased by 3.0% (range: 11%-21%) in the rituximab group, while it increased by 2.0% (range: 14%-25%) in the control group (p = 0.025). Similarly, diffusing capacity of carbon monoxide (DLCO) decreased by 3.0% (range: 10%-12%) in the rituximab group, whereas it increased by 4.5% (range: 30%-36%) in the control group (p = 0.046). Mixed model analysis, adjusting for ILD duration, baseline DLCO, systemic sclerosis, pulmonary hypertension, and prednisone use, did not reveal significant differences in FVC or DLCO between the groups at 6 months or 1 year. The average daily prednisone dose score decreased post-treatment in the rituximab group, while it remained unchanged in the control group (p = 0.017).

Primary Outcomes

Lung function (FVC and DLCO): Rituximab versus cyclophosphamide: Rituximab demonstrated similar efficacy to cyclophosphamide in improving lung function. One RCT reported a mean improvement in FVC of 4.2% with rituximab compared to 2.6% with cyclophosphamide over 12 months. Another observational study showed stabilization of DLCO in both treatment groups [6,9,10].

Rituximab versus mycophenolate: Studies comparing rituximab with mycophenolate showed no significant differences in lung function outcomes. Both treatments resulted in either stabilization or modest improvements in FVC and DLCO. One cohort study noted a 3.8% improvement in FVC with rituximab compared to 3.5% with mycophenolate [16,17].

Secondary Outcomes

Survival rates: Survival data were limited, but one RCT suggested a trend towards better survival with rituximab compared to cyclophosphamide at 24 months follow-up, although this was not statistically significant.

Quality of life: Patient-reported outcomes indicated improvements in quality of life measures with rituximab, comparable to those seen with cyclophosphamide and mycophenolate [9,17].

Adverse Events and Safety Profiles

Rituximab was found to be associated with fewer severe adverse events compared to cyclophosphamide. Common side effects included mild infusion reactions and infections, but no new safety signals were identified [1,3]. Cyclophosphamide had a higher incidence of severe adverse events, including leukopenia, hemorrhagic cystitis, and an increased risk of serious infections [3,8]. Mycophenolate was found to be generally well-tolerated, with gastrointestinal symptoms being the most common adverse events, but overall, fewer severe side effects than cyclophosphamide [3,15,22].

Modified Rodnan Skin Score Comparison

Rituximab demonstrated a notable improvement in mRSS. In several studies, patients treated with rituximab showed a significant reduction in mRSS over a treatment period of 24 to 48 weeks [23]. The mean difference in mRSS reduction with rituximab was −7.01 (95% CI, −11.46 to −2.56), reflecting a substantial decrease in skin fibrosis compared to baseline measurements [22,23]. For cyclophosphamide, the patients treated with cyclophosphamide also exhibited improvements in mRSS, although the extent of the reduction was generally less pronounced than that of rituximab [5]. In comparative studies, the decrease in mRSS with cyclophosphamide was evident but did not reach the same level of statistical significance as that observed with rituximab [6]. For mycophenolate, its impact on mRSS was mixed, with some studies indicating modest improvements while others showed minimal change [15]. Overall, mycophenolate appeared to be less effective in reducing mRSS than rituximab and cyclophosphamide, although it was better tolerated by patients [16,17].

Markers of Disease Progression (HRCT Findings)

Rituximab consistently showed the most significant improvements in HRCT findings, with many patients experiencing stabilization or partial resolution of lung fibrosis and ground-glass opacities [7]. Cyclophosphamide demonstrated moderate effectiveness in stabilizing HRCT findings, with fewer patients showing further progression of ILD compared to mycophenolate but with less improvement than rituximab [10]. Mycophenolate was found to be effective in preventing ILD progression, as seen on HRCT, with stabilization of disease markers, although significant improvements in fibrosis were less common compared with rituximab [14,15].

Subgroup Analyses

Subgroup analyses indicated that patients with early-stage SSc-ILD and those with higher baseline lung function might benefit more from rituximab than those with advanced disease [11]. However, the data were limited, and further research is needed to confirm these findings.

Summary of Findings

Rituximab appears to be an effective and safer alternative to cyclophosphamide for treating SSc-ILD, showing comparable efficacy in improving lung function with fewer severe adverse events [24]. When compared to mycophenolate, rituximab demonstrated similar lung function outcomes and a favorable safety profile [19]. These findings suggest that rituximab is a viable option for SSc-ILD treatment, particularly for patients who cannot tolerate cyclophosphamide or those with early-stage disease [3,6].

Discussion

The treatment of ILD in SSc remains a significant challenge due to the heterogeneity of the disease and the variable response to therapies [14]. This systematic review aimed to compare the efficacy of RTX, CYC, and MMF in managing SSc-ILD, focusing on outcomes related to pulmonary function, quality of life, and adverse events.

Efficacy of Rituximab

RTX, a monoclonal antibody targeting CD20-positive B cells, has shown promise in the treatment of various autoimmune diseases, including SSc-ILD. Several studies included in this review demonstrated that RTX significantly improved FVC and the mRSS [14]. For instance, a study by Lepri et al. (2016) found that RTX treatment resulted in a mean increase in FVC and a significant improvement in mRSS over 24 to 48 weeks. This suggests that RTX not only helps in stabilizing lung function but also has a beneficial effect on skin fibrosis, which is a major concern in SSc patients [11,12].

Moreover, the safety profile of RTX appeared favorable compared to other immunosuppressive agents. The incidence of severe adverse events was lower in patients receiving RTX than those treated with CYC, as indicated in multiple studies [9,10]. For example, an RCT comparing RTX and CYC found fewer adverse events in the RTX group, with participants reporting fewer infections and less leukopenia [23-25].

Efficacy of Cyclophosphamide

CYC, an alkylating agent, has been widely used in the treatment of SSc-ILD due to its potent immunosuppressive properties [10]. The evidence from this review indicates that CYC is effective in improving pulmonary function and reducing breathlessness in SSc-ILD patients [24]. A notable study showed that CYC treatment led to a 2.83% reduction in the decline of %FVC predicted at 12 months compared to placebo, highlighting its role in preserving lung function over time [8-10].

However, the use of CYC is associated with significant toxicity [3]. Patients receiving CYC were more likely to experience adverse events such as leukopenia, infections, and gastrointestinal symptoms [1,3,13]. These side effects often necessitate dose adjustments or discontinuation of therapy, limiting the long-term use of CYC in SSc-ILD patients [26]. Furthermore, the comparison between CYC and MMF revealed that CYC had a higher risk of premature discontinuation due to adverse events, underscoring the need for careful patient monitoring during treatment [16,19].

Efficacy of Mycophenolate

MMF, a compound that inhibits inosine monophosphate dehydrogenase, has emerged as a viable alternative to CYC for the treatment of SSc-ILD [15]. The studies included in this review consistently demonstrated that MMF is effective in stabilizing or improving pulmonary function [19]. For example, one study reported a mean increase in FVC and DLCO in patients treated with MMF, indicating its potential in maintaining lung health in SSc-ILD patients [15-17].

Compared to CYC, MMF was associated with a lower incidence of adverse events, making it a more tolerable long-term treatment option. The review highlighted that patients on MMF experienced fewer hematologic and gastrointestinal side effects than those on CYC [16]. Additionally, MMF was favored over CYC in terms of treatment discontinuation rates, suggesting better patient adherence and satisfaction with MMF therapy [25].

Comparative Efficacy and Safety

When comparing the three agents, RTX appears to offer a favorable balance between efficacy and safety [6]. The significant improvements in FVC and mRSS, coupled with a lower incidence of severe adverse events, position RTX as a promising therapy for SSc-ILD [12]. However, the higher cost and limited availability of RTX may restrict its widespread use, especially in resource-limited settings [11,12].

CYC remains a potent option for short-term treatment, particularly in patients with severe and rapidly progressive ILD [8]. Its efficacy in improving pulmonary function is well-documented, but the high toxicity profile necessitates close monitoring and may limit its long-term use [10].

MMF stands out as a well-tolerated alternative to CYC, offering comparable efficacy with a better safety profile [16]. The lower incidence of adverse events and better patient adherence make MMF an attractive option for long-term management of SSc-ILD [15].

Quality of Life and Functional Outcomes

Quality of life is a critical consideration in the management of SSc-ILD, as the disease significantly impacts patients' daily functioning and overall well-being. The studies reviewed indicated that RTX and MMF had a positive impact on quality-of-life measures, such as the Transition Dyspnea Index and the Health Assessment Questionnaire-Disability Index [8]. Patients reported improvements in breathlessness, disability, and overall physical function, which are important factors in the holistic management of SSc-ILD [17].

CYC also showed improvements in quality-of-life measures, but the high rate of adverse events often counterbalanced these benefits [3,9]. The need for frequent hospital visits and the risk of severe side effects can adversely affect patients' quality of life, making CYC less favorable for long-term treatment compared to RTX and MMF [17].

Adverse Events and Long-Term Safety

Adverse events are a major concern in the treatment of SSc-ILD, as the immunosuppressive nature of these therapies increases the risk of infections and other complications [3]. RTX was associated with a lower incidence of severe adverse events compared to CYC, making it a safer option for long-term therapy. The most common adverse events reported with RTX were mild to moderate infections, which were manageable with standard care [1].

CYC, on the other hand, had a higher incidence of leukopenia, gastrointestinal symptoms, and other severe adverse events [3,5]. These complications often necessitate dose reductions or discontinuation of therapy, limiting its long-term use. The risk of malignancies with long-term CYC use also raises concerns, emphasizing the need for safer alternatives [10].

MMF had a favorable safety profile, with fewer hematologic and gastrointestinal side effects than CYC [15]. The lower risk of serious adverse events makes MMF a more suitable option for long-term management, especially in patients who cannot tolerate CYC [16].

Limitations of the Review

This systematic review has several limitations that should be considered when interpreting the findings. Firstly, the heterogeneity of the included studies in terms of study design, patient populations, and outcome measures poses challenges in making direct comparisons between the therapies. The variations in treatment protocols, follow-up durations, and definitions of clinical outcomes can influence the results and limit the generalizability of the findings. Secondly, the relatively small sample sizes and the lack of large-scale RCTs for some therapies, particularly RTX, limit the strength of the evidence. Larger, well-designed studies are needed to confirm the efficacy and safety of RTX in SSc-ILD and to establish its role in clinical practice. Thirdly, the potential for publication bias cannot be excluded, as studies with positive outcomes are more likely to be published than those with negative or inconclusive results. This bias can skew the overall findings and overestimate the benefits of the therapies. Finally, the lack of long-term follow-up data for some studies limits the ability to assess the sustained efficacy and safety of the therapies. Long-term studies are essential to understand the chronic nature of SSc-ILD and the impact of these treatments on disease progression and patient outcomes over extended periods.

Future Directions and Clinical Implications

The findings of this systematic review highlight the need for personalized treatment approaches in SSc-ILD, considering the individual patient's disease characteristics, treatment response, and tolerance to therapy. Future research should focus on identifying biomarkers that can predict treatment response and guide therapy selection, allowing for more tailored and effective management of SSc-ILD. Large-scale, multicenter RCTs with long-term follow-up are needed to provide robust evidence on the comparative efficacy and safety of RTX, CYC, and MMF. These studies should incorporate standardized outcome measures and consider patient-reported outcomes to capture the full impact of these therapies on patients' quality of life and daily functioning [7,9,15].

In clinical practice, a multidisciplinary approach involving rheumatologists, pulmonologists, and other specialists is essential for the comprehensive management of SSc-ILD [2]. Close monitoring of patients for adverse events and regular assessment of pulmonary function and quality of life are crucial to optimizing treatment outcomes and minimizing complications [3].

The use of combination therapies, such as RTX with MMF, may offer synergistic benefits and improve treatment outcomes in SSc-ILD [17]. Further research is needed to explore the potential of combination regimens and to determine the optimal dosing and duration of therapy. Personalized treatment approaches, guided by robust clinical evidence and patient preferences, are essential for optimizing the management of SSc-ILD and improving patient outcomes.

## Conclusions

In conclusion, this systematic review provides valuable insights into the comparative efficacy and safety of RTX, CYC, and MMF in the treatment of SSc-ILD. RTX appears to offer a favorable balance between efficacy and safety, making it a promising option for long-term management. CYC remains a potent short-term treatment but is limited by its high toxicity profile. MMF stands out as a well-tolerated alternative with comparable efficacy to CYC. While these findings are promising, further large-scale RCTs are needed to validate these results and explore the long-term outcomes of RTX treatment. A multidisciplinary approach, regular monitoring, and tailored therapeutic strategies remain essential for managing patients with SSc-ILD.
